# Multicenter Comparison of Modern Plating Options in Fibula, Scapula, and Osteocutaneous Radial Forearm Free Flaps

**DOI:** 10.1002/ohn.70225

**Published:** 2026-03-26

**Authors:** Craig A. Bollig, Sahithi Reddi, Amit Walia, Patrik J. Pipkorn, Ryan S. Jackson, Sidharth V. Puram, Jason T. Rich, Randal C. Paniello, Jose P. Zevallos, Madelyn N. Stevens, C. Burton Wood, Sarah L. Rohde, Kevin Sykes, Andres Bur, Margaret E. Wieser, Tabitha L. I. Galloway, Patrick Tassone, Tyler Pluchino, Jeffrey B. Jorgensen, Kiran Kakarala

**Affiliations:** ^1^ Head and Neck Surgery and Communication Sciences Rutgers Robert Wood Johnson Medical School Brunswick New Jersey USA; ^2^ Otolaryngology Head and Neck Surgery Washington University St. Louis Missouri USA; ^3^ Otolaryngology Head and Neck Surgery University of Pittsburgh Medical Center Pittsburgh Pennsylvania USA; ^4^ Otolaryngology Head and Neck Surgery Case Western Reserve University School of Medicine Cleveland Ohio USA; ^5^ Otolaryngology Head and Neck Surgery Tennessee Health Science Center Memphis Tennessee USA; ^6^ Otolaryngology Head and Neck Surgery Vanderbilt University Medical Center Nashville Tennessee USA; ^7^ Heath and Wellness Center Baylor Scott and White Heath and Wellness Center Dallas Texas USA; ^8^ Otolaryngology Head and Neck Surgery University of Kansas Medical Center Kansas City Kansas USA; ^9^ Otolaryngology Head and Neck Surgery University of Kentucky Lexington Kentucky USA; ^10^ Otolaryngology Head and Neck Surgery University of Missouri School of Medicine Columbia Missouri USA; ^11^ Otolaryngology Head and Neck Surgery PRISMA Health Greenville South Carolina USA

**Keywords:** computer aided design (CAD), hardware complications, microvascular reconstruction, osseous free flap, virtual surgical planning (VSP)

## Abstract

**Objectives:**

Evaluate the association of plating strategies with hardware removal and operative time, after adjusting for relevant clinical variables.

**Study Design:**

Retrospective Review.

**Setting:**

Multiple Academic Medical Centers.

**Methods:**

Patients undergoing osseous free flap reconstruction stratified based on the plating strategy [conventional plating with a hand‐bent plate, precontoured plate, and virtual surgical planning (VSP) with patient‐specific hardware and cutting guides]. Variables associated with hardware removal were analyzed using multivariable logistic regression. To evaluate the effect of plating strategy on operative time, a multivariable linear regression model was created. Adjusted odds ratios (aOR) with 95% confidence intervals (CI) were calculated.

**Results:**

1022 patients were analyzed: 650 (63.6%) conventional plate, 163 (15.9%) precontoured plate, and 209 (20.5%) VSP. Compared to conventional plating (aOR: 1.00), VSP was independently associated with lower odds of hardware removal (aOR: 0.39, 95% CI: 0.23‐0.68), while there was no difference with precontoured plates (aOR: 0.84, 95% CI: 0.52‐1.38). Compared to conventional plates, precontoured plates were associated with a reduction in operative time of 31.13 minutes (95% CI: 2.93‐59.33), and VSP was associated with a reduction in 76.87 minutes (95% CI: 50.15‐103.57), adjusting for flap type and defect length.

**Conclusions:**

This multi‐institutional study demonstrates that head and neck osseous reconstruction with customized, patient‐specific hardware and VSP is associated with a lower rate of hardware removal and shorter operative time compared to precontoured or hand‐bent hardware. Additionally, cases utilizing precontoured hardware were associated with shorter operative time, but similar rates of hardware removal compared to conventional plates.

Over the last several decades, osseous free flaps have become the preferred option to restore form and function for many major head and neck bony defects.^1^, ^2^, ^3^ The standard of care for securing the osseous bone segments are titanium plates that are bent to align with the defect and fixated to adjacent native bone.^3^, ^4^, ^5^, ^6^ Conventional reconstructive techniques involve an intraoperative “free‐hand” approach to performing osteotomies during cancer resection, alignment and contouring of the titanium plate, shaping of the bone graft segments, and estimating angles of osteotomies of the donor bone segments.^3^ This process reflects the “art” of reconstructive surgery, and can be time‐consuming with variable accuracy, particularly with less experienced surgical teams.^7^ In recent years technological advancements have occurred that offer alternatives to conventional reconstruction with hand bent plates.^8^ Precontoured mandible reconstruction plates with multiple predetermined sizes have been designed to fit most mandibles and are readily available through the major craniomaxillofacial medical device manufacturers.^8^ Additionally, patient‐specific customized plates can be crafted with computer‐aided design and computer‐aided manufacturing (CAD/CAM) based on preoperative radiographic osseous morphology, as well as patient‐specific 3D models and cutting guides for osteotomies.^9^ The length, shape, thickness, number, and angulation of screw holes can all be matched to reflect the patient′s unique bony contour.^9^ Preoperatively, there is a short, computerized planning session with surgeons and technicians where every step of this process is planned and designed. This approach may also be referred to as virtual surgical planning (VSP), and use has been increasing among head and neck reconstructive surgeons.^10^, ^11^

There has been considerable interest in investigating potential advantages of these emerging technologies in terms of improved patient outcomes, reduction in operative time, or reduced complications as a means to justify the increased costs.^12^, ^13^, ^14^, ^15^, ^16^ Existing literature has consistently shown that CAD/CAM and the use of precontoured plates are associated with improved accuracy and reduction in operative time compared to conventional reconstruction.^17^, ^18^, ^19^, ^20^ The frequency of complications is relatively well‐described in the literature, but evidence is generally restricted to single‐institution reviews with a relatively small number of patients.^17^, ^18^, ^19^, ^20^, ^21^ Additionally, there is a paucity of literature on outcomes of VSP and precontoured plates in scapula free flaps (SFF) and osteocutaneous radial forearm free flaps (OCRFFF) because most studies are overwhelmingly composed of fibula free flaps (FFF).^20^, ^22^

Further delineating potential differences in outcomes between plating strategies in a multi‐institutional cohort composed of OCRFFF, FFF, and SFF would be valuable to head and neck reconstructive surgeons and their patients. Therefore, our primary objective with this study was to evaluate the association of reconstructive plating strategy (conventional reconstruction, precontoured plate, and VSP) with hardware removal using a multi‐institutional cohort of patients undergoing reconstruction of a major osseous head and neck defect with a FFF, OCRFFF, or SFF. A secondary objective was to assess the association of plating strategy on operative time.

## Methods

After the project was designed, institutional review board approval was obtained at: the University of Missouri‐Columbia, the University of Kansas Medical Center, Vanderbilt University Medical Center, Washington University in St. Louis, University of Louisville Research Foundation, and Rutgers Robert Wood Johnson University Hospital in New Brunswick. A retrospective cohort of patients was generated who underwent reconstruction of a head and neck defect with a FFF, OCRFFF, or SFF over a continuous timeframe between January 2005 and December 2019. Patients were excluded if data on clinical demographics, plating strategy, or hardware removal was missing. Prior to data collection, a power analysis was performed to determine an adequate sample size. Assuming hardware removal rate of 30% (conventional reconstruction) and 15% (VSP), power of 0.9, type 1 error rate of 0.05, and a 3 to 1 distribution of conventional reconstruction to VSP, a sample size of at least 249 (conventional reconstruction) and 83 (VSP) was needed.

Patients' electronic medical records and/or pre‐existing institutional flap registries were used to obtain clinical information. Patients were stratified based on the reconstructive plating strategy. Baseline patient characteristics included age, sex, race, Charlson‐Deyo Comorbidity Class (CDCC), diagnosis of diabetes mellitus, current tobacco use, current alcohol use, previous or adjuvant radiation therapy (RT), previous or adjuvant chemoradiotherapy (CRT), bony defect location, indication for reconstruction, bone defect length, free flap type, acute surgical site infection (SSI), acute fistula, and duration of follow‐up. Our primary outcome was the rate of hardware removal (failure). Variables between treatment groups were then compared using the chi‐squared test or Fisher's exact test for categorical variables. Continuous variables were analyzed using analysis of variance (ANOVA) or non‐parametric tests depending on the normality of distribution. The association of plating strategy with hardware removal was evaluated using logistic regression. Variables with *P* < .05 on univariable testing were included in a multivariable logistic regression model along with other clinically relevant risk factors. These were selected a priori. Adjusted odds ratios (aOR) and associated 95% confidence intervals (CI) were calculated for each multivariable model.

To investigate our secondary objective of the association of reconstructive strategy on operative time, a multivariable linear regression model was created that adjusted for free flap type and bone defect length in the subset of patients in whom operative time was available. Because operative efficiency improved over the study period, this was performed in the subset of patients during the end of the study period (between 2012‐2019). For all analyses, the threshold for statistical significance was set at *P* < .05. SPSS v29 software was used for data analysis (SPSS Inc, an IBM Company).

## Results

There were 1022 patients remaining after exclusions with a mean follow‐up of 29.4 (30.3) months. Of these, 650 (63.6%) underwent conventional reconstruction with a hand‐bent plate, 163 (15.9%) with a precontoured plate alone, and 209 (20.5%) with a CAD/CAM customized plate and cutting guides. Table 1 describes the baseline patient characteristics for each group. Overall, these groups were similar with respect to: age, sex, race, diagnosis of diabetes mellitus, current tobacco use, previous RT and CRT, adjuvant RT, defect location, and mean defect length. There were similar rates of acute postoperative fistulas between groups. There were clinically relevant differences between groups in the following variables: CDCC, current alcohol use, adjuvant CRT, reconstructive indication, and osseous free flap type. There were significant differences in CDCC between groups with the following distribution of CDCC ≥ 3: CAD/CAM custom plates (n = 58, 27.8%), precontoured plates (n = 35, 21.5%), conventional reconstruction (n = 375, 57.7%), *P* < .001. CAD/CAM had a higher proportion of current alcohol users (n = 107, 51.2%) versus precontoured plates (n = 66, 40.5%) or conventional plates (n = 228, 35.1%), *P* < .001. The conventional reconstruction cohort had a higher proportion of patients receiving adjuvant CRT (n = 176, 27.1%) versus precontoured plates (n = 32, 19.6%) or CAD/CAM plates (n = 43, 20.6%), *P* = .046. There were significant differences between groups in the indications for reconstructions as follows: conventional reconstruction (malignancy [n = 552, 84.9%] and ORN [n = 60, 10.2%]), precontoured plate (malignancy [n = 122, 74.8%] and ORN [n = 29, 17.8%]), and CAD/CAM plate (malignancy [n = 146, 69.9%] and ORN [n = 46, 22.0%]), *P* < .001. Finally, there were significant differences in the distribution of osseous donor sites between groups: conventional reconstruction (FFF [n = 311, 47.8%], OCRFFF [n = 230, 35.4%], and SFF [n = 109, 16.8%]), precontoured plate (FFF [n = 75, 46.0%], OCRFFF [n = 73, 44.8%], and SFF [n = 15, 9.2%]), and CAD/CAM plate (FFF [n = 124, 59.3%], OCRFFF [n = 73, 34.9%], and SFF [n = 12, 5.7%]) *P* < .001.

**Table 1:** Baseline Characteristics

Variable	Conventional plating N = 650	Precontoured hardware N = 163	Virtual surgical planning N = 209	*P* value
Mean age (SD), years	60.9 (14.4)	60.0 (15.4)	60.8 (14.0)	.996
Sex				.303
Male	419 (64.5%)	111 (68.1%)	146 (69.9%)	
Female	231 (35.5%)	52 (31.9%)	63 (30.1%)	
Race				.085
White	573 (88.2%)	149 (91.4%)	177 (84.7%)	
Black	60 (9.2%)	13 (8.0%)	21 (10.0%)	
Other/not recorded	17 (2.6%)	1 (0.6%)	11 (5.3%)	
CDCC 0	162 (24.9%)	63 (38.7%)	96 (45.9%)	<.001
CDCC 1	38 (5.8%)	34 (20.8%)	32 (15.3%)	
CDCC 2	75 (11.5%)	31 (19.0%)	23 (11.0%)	
CDCC ≥ 3	375 (57.7%)	35 (21.5%)	58 (27.8%)	
Diabetes mellitus	96 (14.8%)	26 (16.0%)	31 (14.8%)	.929
Current tobacco use	226 (34.8%)	64 (39.3%)	69 (33.0%)	.434
Current alcohol use	228 (35.1%)	66 (40.5%)	107 (51.2%)	<.001
Prior RT	178 (27.4%)	53 (32.5%)	68 (32.5%)	.221
Prior CRT	120 (18.5%)	34 (20.9%)	33 (15.8%)	.448
Adjuvant RT	295 (45.4%)	60 (36.8%)	89 (42.6%)	.137
Adjuvant CRT	176 (27.1%)	32 (19.6%)	43 (20.6%)	.046
Prior or adjuvant RT	449 (69.0%)	109 (66.9%)	155 (74.2%)	.258
Location				
Mandible	597 (91.8%)	157 (96.3%)	194 (92.8%)	.724
Maxilla	53 (8.2%)	6 (3.7%)	15 (7.2%)	
Indication				<.001
Malignancy	552 (84.9%)	122 (74.8%)	146 (69.9%)	
ORN	66 (10.2%)	29 (17.8%)	46 (22.0%)	
Benign tumor/trauma	32 (4.9%)	12 (7.4%)	17 (8.1%)	
Mean defect length cm, (SD)	7.4 (2.8)	6.9 (2.5)	7.7 (2.7)	.284
Acute postoperative fistula	75 (11.5%)	19 (11.7%)	27 (12.9%)	.863
Osseous flap type				<.001
FFF	311 (47.8%)	75 (46.0%)	124 (59.3%)	
OCRFFF	230 (35.4%)	73 (44.8%)	73 (34.9%)	
SFF	109 (16.8%)	15 (9.2%)	12 (5.7%)	

Abbreviations: CDCC, Charlson Deyo Comorbidity Class; CRT, chemoradiotherapy; OCRFFF, osteocutaneous radial forearm free flap; ORN, osteoradionecrosis; RT, radiation therapy; SSI, surgical site infection.

**Figure 1:**
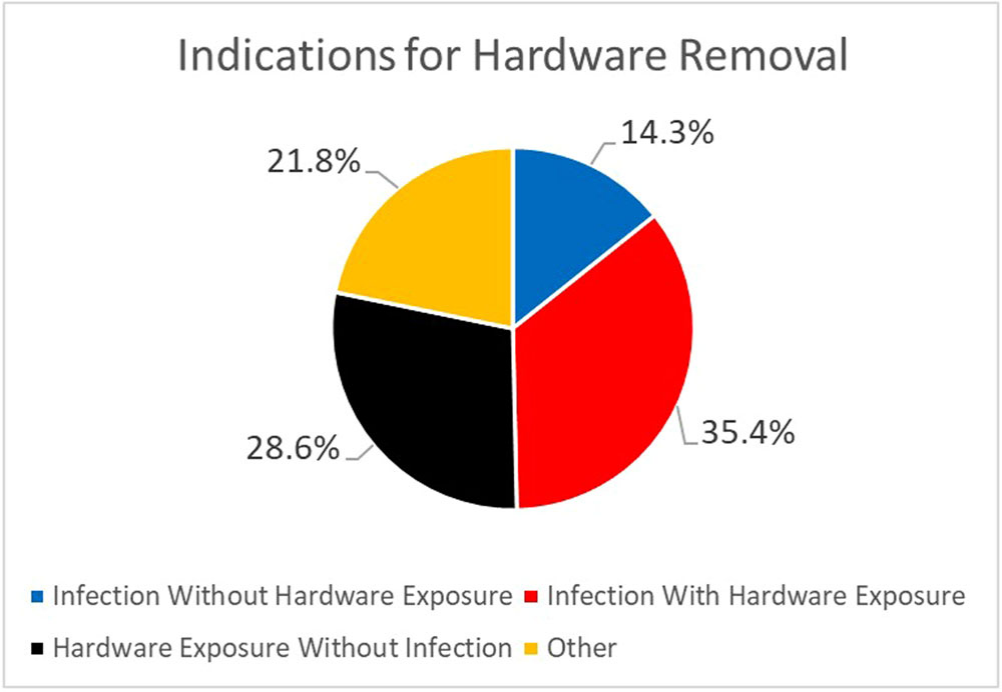
Pie chart detailing the reported indications for hardware removal.

**Figure 2:**
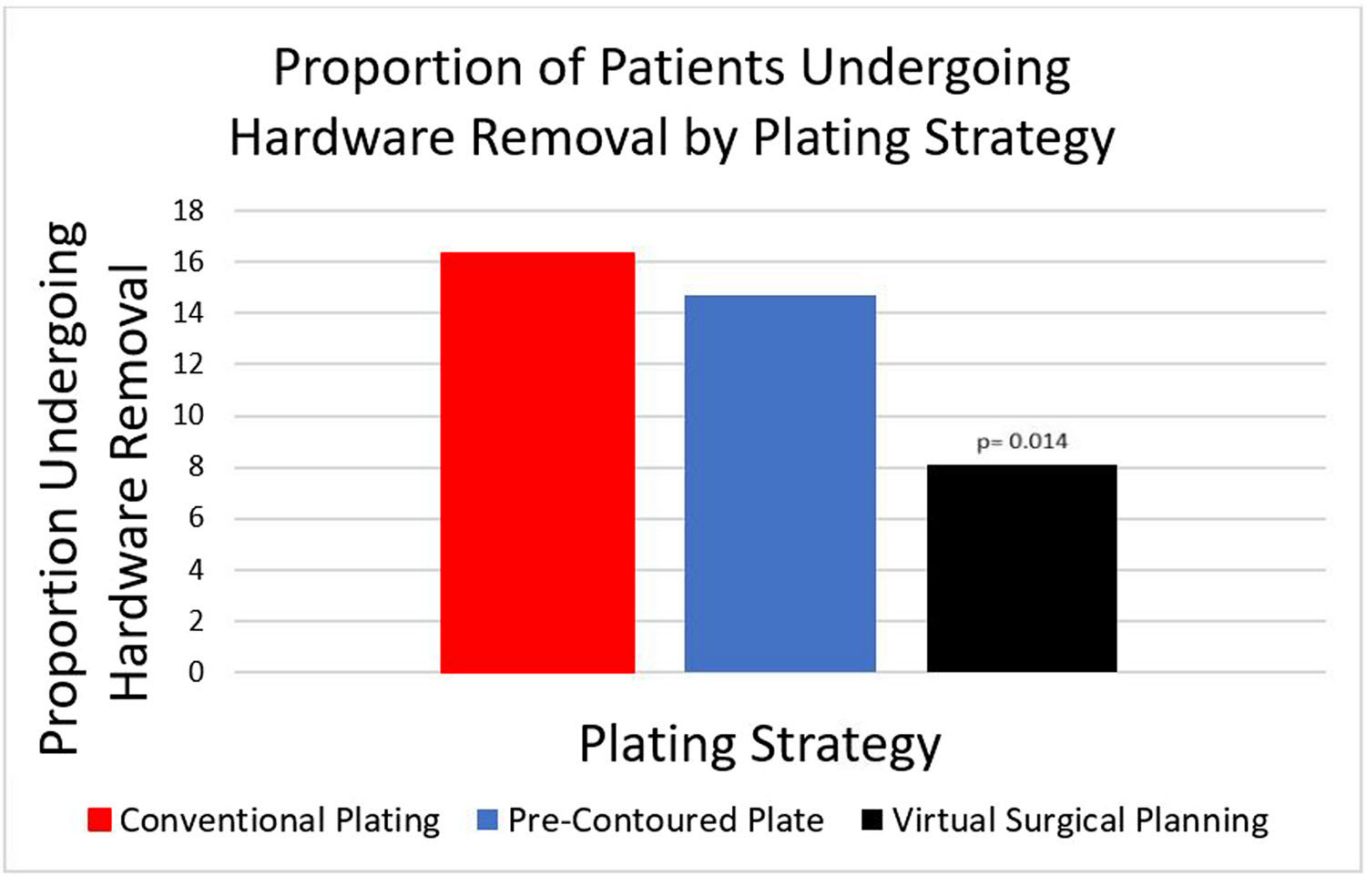
Chart comparing the proportion of patients undergoing hardware removal by plating strategy: conventional plating, precontoured plate, virtual surgical planning.

Overall, 147 (14.4%) patients experienced hardware failure and underwent removal. Figure 1 details the indications for hardware removal: 52 (35.4%) hardware exposure with chronic infection, 42 (28.6%) hardware exposure without infection, 21 (14.3%) chronic infection without hardware exposure, and 32 (21.8%) for other reasons/not reported. Figure 2 depicts the prevalence of hardware removal (failure) with each reconstructive strategy. Traditional hand‐bent plates were associated with the highest rate of hardware removal (n = 106, 16.3%), followed by precontoured plates (n = 24, 14.7%), and CAD/CAM custom plates (n = 17, 8.1%). Table 2 describes the results of the multivariable analyses that controlled for acute postoperative fistulas (aOR: 3.27, 95% CI: 2.09‐5.13), diabetes, current tobacco use, preoperative and/or adjuvant RT (aOR: 1.64, 95% CI: 1.02‐2.63), and free flap donor site (SFF, aOR: 1.00; FFF, aOR: 2.95, 95% CI: 1.47‐5.93; and OCRFFF, aOR: 2.55, 95% CI: 1.24‐ 5.26). Adjusting for these clinically relevant variables, VSP was independently associated with the lower odds of hardware removal (aOR: 0.39, 95% CI: 0.23‐0.68) compared to conventional reconstruction (aOR: 1.00), while there was no difference with precontoured plates (aOR: 0.84, 95% CI: 0.52‐1.38).

**Table 2:** Clinical Factors Associated With Hardware Removal on Multivariable Analyses

Variable	Adjusted odds ratio	95% Confidence interval
Plating strategy		
Conventional plating	1.00	[Reference]
Precontoured plate	0.84	[0.52‐1.38]
Virtual surgical planning	0.39	[0.23‐0.68]
Osseous free flap type		
Scapula free flap	1.00	[Reference]
Fibula free flap	2.95	[1.47‐5.93]
OCRFFF	2.55	[1.24‐5.26]
Acute postoperative fistula	3.27	[2.09‐5.13]
History of radiation therapy	1.64	[1.02‐2.63]
Diabetes mellitus	1.00	[0.60‐1.67]
Current tobacco use	0.96	[0.66‐1.40]
Length of bone defect	1.04	[0.99‐1.09]

Abbreviation: OCRFFF, osteocutaneous radial forearm free flap.

Mean operative time varied significantly by reconstructive strategy. Cases utilizing VSP were associated with the shortest mean operative time (497.5 minutes, SD 142.8), followed by precontoured plate (563.1 minutes, SD 148.2), and conventional plates (625.2 minutes, SD 149.7), *P* < .001 for all comparisons. Cases performed during the first part of the study period (2005‐2011) were associated with increased operative time (712.6 minutes, SD 160.8) versus those performed during 2012 to 2019, (552.5 minutes, SD 132.2), *P* < .001.

Table 3 details the results of the multivariable linear regression model that adjusted for cofounding variables that also contribute to operative time‐osseous free flap type and bone defect length. This was performed in the subset of patients undergoing surgery between 2012 and 2019, to adjust for the improvement in operative efficiency that was observed during the study period. In this model, compared to conventional hand‐bent plates, precontoured plates were associated with an estimated reduction in operative time of 31.13 minutes (95% CI: 2.93‐59.33), and VSP was associated with a reduction in 76.86 minutes (95% CI: 50.15‐103.57). Operative time increased an estimated 9.85 minutes (95% CI: 5.30‐14.40) per additional centimeter of bone defect length. Compared to FFF, OCRFFF were associated with a reduction in operative time of 29.58 minutes (95% CI: 6.20‐52.96), while SFF were associated with an increase in operative time of 83.45 minutes (95% CI: 47.17‐119.73).

**Table 3:** Multivariable Linear Regression Analysis of Clinical Factors Associated With Operative Time

Variable	Estimate (minutes)	95% Confidence interval
Plating strategy		
Conventional plating	[Reference]	
Precontoured plate	−31.13	[−59.33 to −2.93]
Virtual surgical planning	−76.86	[−103.57 to −50.15]
Osseous free flap type		
Fibula free flap	[Reference]	
Scapula free flap	83.45	[47.17‐119.73]
OCRFFF	−29.58	[−6.20 to −52.96]
Length of bone defect (per 1 mm)	9.85	[5.30‐14.40]

Abbreviation: OCRFFF, osteocutaneous radial forearm free flap.

## Discussion

In this multi‐institutional analysis of over 1000 patients undergoing osseous reconstruction of the head and neck, we compare hardware removal rates as well as operative times between osseous plating strategies. While precontoured hardware had similar rates of hardware removal compared to conventional plating techniques, we found significantly lower rates of hardware removal in cases where VSP was utilized with customized CAD/CAM plates and cutting guides. Additionally, we found a significant reduction in operative time with both precontoured hardware and VSP compared to conventional plating; with significantly lower operative times in VSP versus precontoured plates.

While osseous free flap loss at most tertiary care centers is rare, hardware complications including failure that necessitates removal remain quite common.^23^, ^24^, ^25^, ^26^, ^27^, ^28^, ^29^ Potential causative factors for hardware removal that have been reported include: chronic infection related to the plate, non‐healing wound with or without plate exposure, chronic pain without other identifiable sources, loose hardware, and hardware exposure without evidence of infection.^28^, ^29^ The prevalence of hardware removal in this cohort was 14.4%, which is toward the mid‐lower end of the reported range in the literature (7.5%‐27%).^23^, ^25^, ^26^, ^28^, ^30^

After adjusting for variables that have previously been shown to be associated with hardware complications or may affect wound healing, we found that CAD/CAM plates were independently associated with the lowest rates of hardware removal. Overall, results in the literature have been mixed regarding the association of VSP with hardware complications. However, most series have been underpowered to detect clinically meaningful differences in complication rates.^17^, ^18^, ^19^, ^31^ In four systematic reviews with meta‐analyses comparing VSP to traditional plate bending, the sample size range for patients undergoing VSP were (5‐26), (5‐70), (7‐52), 5‐38).^17^, ^18^, ^19^, ^31^ Much less is reported in the literature about outcomes of precontoured plates, but in a comparative study of pre‐bent plates versus customized CAD/CAM plates with 142 patients, McCann et al found lower rates of hardware removal in patients with CAD/CAM plates (20.2%) versus standardized precontoured plates (5.6%).^20^ As a potential explanation for lower rates of hardware failure in CAD/CAM plates, surgical accuracy has been shown to be associated with the development of hardware complications.^32^ Davies et al demonstrated that a plate‐bone graft distance of >1 mm was associated with 3.5 greater odds of plate exposure in their review of 94 patients in 2022.^32^ Similarly, May et al demonstrated significantly higher bone union rates in VSP (96%) versus conventional methods (80%) as well as a shorter median time to bone union (0.8 years [VSP] vs 1.2 years [conventional methods]).^33^ Other advances in hardware plating systems including the use of locking plates and screws have required less precision in plate adaptation due to the internal/external fixator mechanism as well as lower incidence of screw loosening over time.^34^

Although not evaluated in this study, reconstructive accuracy has been thoroughly compared between techniques. Common accuracy measures have included cephalometric measures (gonial angle, intergonial angle difference, gonion‐gnathion distance, condyle‐gonion distance, and condyle shift), interfragmentary gaps, and plate‐bone graft distance.^7^, ^17^, ^18^, ^19^, ^32^ Results have been mixed in the literature, but there has been a trend toward a higher accuracy rate in reconstructions utilizing VSP.^7^, ^17^, ^18^, ^19^ However, the differences in accuracy measures complicate synthesis of the literature in a meta‐analysis.

Previous literature has consistently demonstrated significant reductions in operative time with the use of VSP.^12^, ^15^, ^16^, ^35^, ^36^, ^37^, ^38^, ^39^ In this cohort, after adjusting for osseous flap type and length of bone defect, VSP was associated with an estimated reduction in operative time by a mean of 76.9 minutes. A 2019 meta‐analysis by Tang et al reported lower operative times in 14 of 15 studies with VSP, with mean differences ranging from 10.0 to 172.7 minutes.^17^ They reported a standardized mean difference of −1.01 in the operative times between VSP and non‐VSP, which translated to a large effect size.^17^ In this cohort, precontoured plates were associated with a reduction in operative time by a mean of 31.1 minutes. Precontoured plates offer the potential time‐saving advantage of avoiding the need to hand‐bend hardware to match the contour of the native bone. However, VSP includes a precontoured plate that is customized to the patient and planned bone grafts as well as cutting guides for the bone grafts as well as the ablation. These additional components may translate into the additional time savings seen with VSP over standardized pre‐bent plates. It is worth noting that not all studies have found reductions in operative time with VSP.^40^, ^41^ It is likely that this difference would be most prominent in less experienced reconstructive surgeons and may not translate to high‐volume surgeons with more clinical experience with osseous reconstruction. Additionally, an important factor to consider is that while VSP has been more commonly utilized over time, there has also been a general trend toward improved operative efficiency over time. In this cohort, we observed a decrease in operative case time during the study period, therefore this was adjusted for in the analysis.

There are limitations of VSP that are important considerations, chiefly increased cost as well as additional time to acquire models, cutting guides, and customized hardware. In an era with skyrocketing health care costs, VSP has appropriately been put under scrutiny to justify its use. In a 2024 systematic review of cost outcomes of VSP in head and neck reconstruction, Xiao et al reported that 14 of 18 cost analyses demonstrated neutral or net cost savings with VSP when factoring in reduced operative time, which has been consistently reported as mentioned above.^42^ Additional potential sources of cost savings that have been reported to be associated with VSP in some series include reductions in duration of hospital stay, fewer medical complications that are associated with increased operative time, and decreased hardware complications.^19^ Given the reduction in operative time as well as decreased complications, we anticipate a reduction in cost would be seen in our cohort, although a formal cost‐effectiveness analysis was not performed. Another important consideration that has been reported with VSP is the potential for increased time to surgery. In a review of 165 patients, Villarme et al reported that utilization of VSP was associated with a significant increase in the mean time to surgery (44 days) versus 31 days for cases without VSP.^43^ Time to surgery has been shown to be independent predictor of survival in head and neck cancer.^44^ Outside of the potential survival impact, additional tumor growth results in a larger oncologic resection, which may have a functional consequence as well. Weighing the potential benefits of VSP versus the limitations needs to be performed on case‐by‐case basis. We noted an increased utilization of VSP during non‐malignant cases (ORN, trauma, and benign tumors), which may reflect less concern of potential increases in time to surgery in that population.

Most existing series on VSP in head and neck reconstruction have overwhelmingly been composed of FFF, while much less is published in SFF and OCRFFF.^20^, ^25^ This cohort was evenly balanced between FFF and non‐FFF (50% of each) and provides evidence that VSP can be successfully utilized in OCRFFF and SFF. This study has several limitations. Selection bias is inherent in retrospective studies, but multivariable models were utilized to assess the impact of known confounding variables. Information on some relevant medical conditions that may affect wound healing were not available for analysis including hypothyroidism and malnutrition. Finally, the rates of malunion and nonunion may differ between plating strategies but were not analyzed due to concerns of under detection.^45^ The study design best suited for that analysis would involve active review of individual patient images and was not feasible in this multi‐institutional setting with this large of a sample size.

## Conclusion

This multi‐institutional study demonstrates that head and neck osseous reconstruction with customized patient‐specific hardware and VSP is associated with a lower rate of hardware removal and shorter operative time compared to precontoured or hand‐bent hardware. Additionally, cases utilizing precontoured hardware were associated with shorter operative time, but similar rates of hardware removal compared to hand‐bent hardware.

## Author Contributions

**Craig A. Bollig**: conception, design, acquisition, analysis, interpretation, drafting, revision, final approval, accountability agreement; **Sahithi Reddi**: acquisition, drafting, revision, final approval, accountability agreement; **Amit Walia**: acquisition, revision, final approval, accountability agreement; **Patrik J. Pipkorn**: design, revision, final approval, accountability agreement; **Ryan S. Jackson**: revision, final approval, accountability agreement; **Sidharth V. Puram**: revision, final approval, accountability agreement; **Jason T. Rich**: revision, final approval, accountability agreement; **Randy C. Paniello**: revision, final approval, accountability agreement; **Jose P. Zevallos**: revision, final approval, accountability agreement; **Madelyn N. Stevens**: acquisition, revision, final approval, accountability agreement; **C. Burton Wood**: design, acquisition, revision, final approval, accountability agreement; **Sarah L. Rohde**: revision, final approval, accountability agreement; **Kevin Sykes**: acquisition, revision, final approval, accountability agreement; **Andres Bur**: revision, final approval, accountability agreement; **Margaret E. Wieser**: acquisition, revision, final approval, accountability agreement; **Tabitha L. I. Galloway**: design, revision, final approval, accountability agreement; **Patrick Tassone**: design, acquisition, revision, final approval, accountability agreement; **Tyler Pluchino**: acquisition, revision, final approval, accountability agreement; **Jeffrey B. Jorgensen**: design, revision, final approval, accountability agreement; **Kiran Kakarala**: design, revision, final approval, accountability agreement.

## Disclosures

### Competing interests

None.

### Funding source

None.
